# A case of hereditary breast and ovarian cancer syndrome of initially presented as cancer of unknown primary with lymph node metastases unveiled by genetic analysis

**DOI:** 10.1007/s13691-023-00652-4

**Published:** 2024-02-09

**Authors:** Juri Yamada, Koji Fukuda, Tae Sugawara, Kenichi Makino, Kazuhiro Shimazu, Taichi Yoshida, Daiki Taguchi, Hanae Shinozaki, Yukihiro Terada, Hiroshi Nanjo, Hiroyuki Shibata

**Affiliations:** 1 Department of Clinical Oncology, Graduate School of Medicine, Akita University, Hondo 1-1-1, Akita, Japan; 2 Department of Obstetrics and Gynecology, Graduate School of Medicine, Akita University, Akita, Japan; 3 Department of Pathology, Akita University Hospital, Akita, Japan

**Keywords:** BRCA1 variant, Cancer of unknown primary, HBOC syndrome, Homologous recombination deficiency, PARP inhibitor

## Abstract

Cancer of unknown primary (CUP) is a heterogeneous disease concept involving various malignant tumors. Understanding its pathophysiology is often difficult, together with its treatment. Here, we present a case of CUP with abdominal lymph node enlargement and elevated carbohydrate antigen 125 levels. It initially resembled a favorable prognosis type similar to ovarian cancer, but metastases were observed in cervical lymph nodes, indicating a somewhat atypical CUP compared to the typical ovarian cancer-like CUP. We identified a germline Breast Cancer 1 (BRCA1) p.L63* variant through a family history inquiry and BRCA analysis, indicating hereditary breast and ovarian cancer syndrome. The patient achieved near-complete remission with platinum-based therapy followed by poly (ADP-ribose) polymerase (PARP) inhibitor. The variant has shown sensitivity in both clinical and pathogenic reports in the ClinVar database of the National Institutes of Health. No clinical studies reported on the efficacy of PARP inhibitors specific to this variant, but our case demonstrated the sensitivity of platinum-based therapy followed by PARP inhibitor. Reports of CUP in hereditary breast and ovarian cancer syndrome are very rare, with only a single report in the literature.

## Introduction

Cancer of unknown primary (CUP) is histologically defined as a metastatic malignancy in which the primary site remains unidentified. The frequency of CUP is reported as 2%–5% of all malignant tumors.

The clinical presentation of CUP differs among patients, with many experiencing poor prognoses. Early dissemination, rapid progression, and unpredictable patterns of metastasis, resulting in a median survival of approximately 8–12 months, are some key characteristics of CUP. However, certain CUP subtypes exhibit improved prognoses, with overall survival of 12–36 months, including midline embryonal tumors, estrogen receptor-positive adenocarcinomas with axillary lymph node metastasis only, adenocarcinomas with peritoneal dissemination and elevated carbohydrate antigen 125 (CA125) levels, male adenocarcinomas with osteoblastic bone metastasis and accompanying prostate-specific antigen elevation, squamous cell carcinomas with cervical lymph node metastasis only, squamous cell carcinomas with inguinal lymph node metastasis only, and tumors with neuroendocrine histology [1].

Hereditary breast and ovarian cancer (HBOC) syndrome is characterized by autosomal dominant inheritance patterns caused by pathogenic variants in the germ line of *Breast Cancer 1/2* (*BRCA1/2*). HBOC syndrome is associated with increased breast, ovarian, pancreatic, and prostate cancer susceptibility [2].

Pathogenic variants in *BRCA1/2* result in defective homologous recombination repair mechanisms. Consequently, tumors harboring pathogenic variants in *BRCA1/2* are sensitive to platinum-based agents and poly ADP-ribose polymerase (PARP) inhibitors.

A sequencing study conducted by Welsh et al. revealed that 24% of 360 cases of primary ovarian, peritoneal, and fallopian tube cancer harbored pathogenic variants in genes associated with the homologous recombination repair pathway, such as BRCA1/2 [3]. Cases with germline variants in genes other than *BRCA1/2* accounted for 6% of all cases examined, representing one-third of patients with pathogenic variants in their germ cell line in their study [3].

Here, we present a case of CUP with adenocarcinoma histology and a germline BRCA1 p.L63* variant, emphasizing the clinical course of this rare presentation and clinical response with platinum-based therapy followed by PARP inhibitor.

## Case presentation

**Fig. 1:**
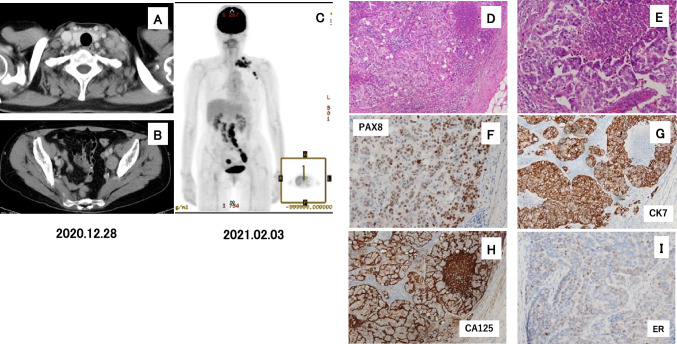
Clinicopathological features. A. Computed tomography (CT) imaging of the cervical region. B. CT imaging of the pelvic region. C. Positron emission tomography—CT imaging. D. Hematoxylin-Eosin (HE) staining of the cervical lymph node metastasis. E. Higher magnificent imaging of D. F. Immunohistochemistry (IHC) of the cervical lymph node metastasis with anti-PAX8 antibody. G. IHC with anti-cytokeratin (CK) 7 antibody. H. IHC with anti-carbohydrate antigen 125 (anti-CA125) antibody. I. IHC with anti-estrogen receptor (ER) antibody.

A 69-year-old female patient presented with left cervical lymphadenopathy and sought medical attention by former doctors in December 2020. The computed tomography (CT) imaging and the magnetic resonance imaging demonstrated cervical and pelvic region lymphadenopathies and myoma uteri (Fig. 1A, B) but with no other tumors in the digestive tract or gynecological organs. The previous gynecologists could not find any gynecological lesions after their examination. The positron emission tomography (PET)—CT scan revealed uptake in the left supraclavicular, left axillary, para-aortic, and left external iliac lymph nodes, with no significant uptake in other organs (Fig. 1C).

The histopathological cervical lymph node examination revealed a moderately to poorly differentiated adenocarcinoma (Fig. 1D, E). No significant medical history findings were noted. Laboratory tests revealed an elevated CA125 level of 2032.8 U/mL.

**Fig. 2:**
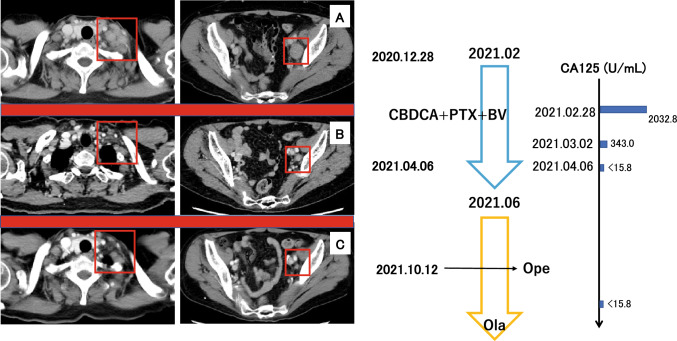
Clinical course of the case. A. CT imaging of the left cervical and intrapelvic lymph node metastases on December 28, 2020. B. CT imaging on April 6, 2021. C. CT imaging on October 12, 2021. Chemotherapy with paclitaxel (PTX), carboplatin (CBDCA), and bevacizumab (BV) was started on February 2021 and followed by olaparib (Ola) on June 2021. The CA125 level was examined on February 28, 2021, March 2, 2021, and April 6, 2021, respectively.

The former doctors diagnosed this disease as a CUP case and the patient was referred to our department in February 2021. Immunohistochemistry (IHC) of the cervical lymph node metastasis was positive for CK7, negative for CK20, positive for CA125, partially positive for PAX8, and partially positive for estrogen receptor (ER) staining, indicating an ovarian primary tumor (Fig. 1F–I). We asked our breast surgeons to examine her breast, but no tumors were detected. We diagnosed this case as occult ovarian and fallopian tube cancers (OCC) by pelvic lymph node swelling, the results of IHC and the elevation of CA125 level. She had the cervical lymph node metastases, and we considered this case was equal to Stage 4B ovarian cancer. Two months were already passed since her first visit to the medical service. The patient started receiving combination chemotherapy with paclitaxel (PTX), carboplatin (CBDCA), and/or bevacizumab (BV) by medical oncologists in February 2021 (Fig. 2A). All target lesions revealed a size reduction after completing six chemotherapy cycles (Fig. 2B, C), and the CA125 level was normalized after 1.5 months of PTX + CBDCA + BV (Fig. 2).

**Fig. 3:**
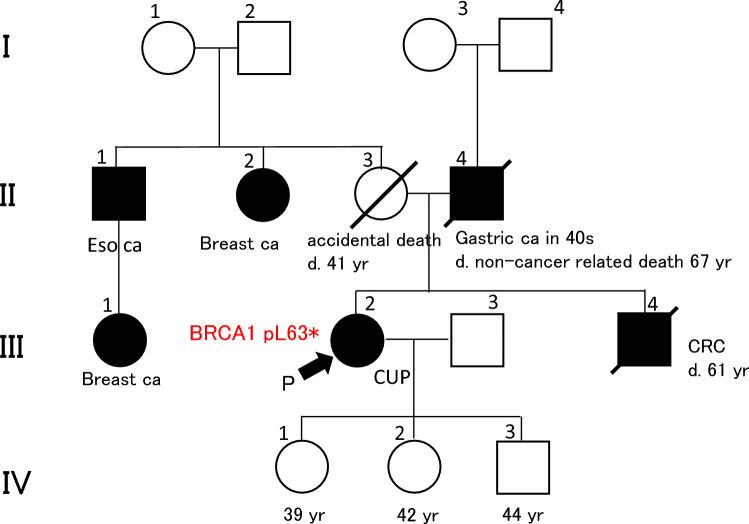
Family tree of this case. P: proband. Eso ca: esophageal cancer, CUP: cancer of unknown primary, CRC: colorectal cancer

Simultaneously, we examined her family history precisely. It revealed an esophageal cancer in the patient’s maternal uncle (Fig. 3, II-1) and breast cancers in her daughter (Fig. 3, III-1) and her maternal aunt (Fig. 3, II-2). The accumulation of breast cancer was indicated to be apparent in the maternal lineage, and HBOC syndrome was suspected. Genetic testing for *BRCA* variants (BRACAnalysis®, Myriad Genetics, Inc., Salt Lake City, Utah, United States) was performed in June 2021, which detected a germline pathogenic variant of BRCA1 p.L63*. Consequently, the patient was diagnosed with HBOC syndrome. The treatment was followed by maintenance therapy with the PARP inhibitor, olaparib (Ola), due to the platinum sensitivity maintained with chemotherapy composed of PTX, CBDCA, and BV. Thereafter, we discussed this case with our gynecologists in our board meeting. The patient underwent bilateral salpingo-oophorectomy, total hysterectomy, and partial omentectomy through laparotomy in October 2021 from the viewing point of risk-reducing salpingo-oophorectomy (RRSO). The pathological examination of the resected specimens revealed no existence of cancer cells according to the sectioning and extensively examining the fimbriated end protocol, indicating a complete chemotherapy response [4]. The primary ovarian cancer origin was presumed to have completely disappeared. Since then, the patient continues Ola treatment, and lymph node metastases in the left supraclavicular, left axillary, para-aortic, and left external iliac regions have decreased under the detectable limit, while maintaining a complete response. The CA125 level remains under the normal limit.

The patient undergoes regular breast screening because of the 72% cumulative risk of breast cancer development by the age of 80 years in *BRCA1* variant carriers. Additionally, genetic counseling and testing are planned as the patient has three children [3].

## Discussion

CUP is a histologically confirmed metastatic malignant tumor with an unidentified primary site of origin. The clinical presentation of CUP varies among patients [1]. Treatment following the NCCN Ovarian Cancer Guidelines is recommended for patients with adenocarcinoma presenting as intraperitoneal tumors or ascites, where ovarian origin cannot be ruled out based on histological findings [1]. Treatment with either PTX plus CBDCA, docetaxel plus CBDCA, or PTX plus CBDCA plus BV is recommended for epithelial ovarian cancer, fallopian tube cancer, primary peritoneal cancer, and rare histological types [1]

This case demonstrated abdominal lymphadenopathy and elevated CA125, as well as metastases in the left cervical and left axillary lymph nodes, which were slightly exceptional from the typical presentations described in the guidelines.

HBOC syndrome was suspected based on the family history, and BRACAnalysis® was performed, revealing her germline variant in BRCA1 c.L63*. BRCA1,

BRCA1 p.L63* variant is a nonsense variant where codon 63 is changed to a stop codon. Hence, a large BRCA1 protein consisting of 1863 amino acids loses the C-terminal portion distant from codon 63. The BRCA1 c.L63* variant, where all of functional domains are lost, results in the loss of BRCA1-PALB2-BRCA2 trimer formation impairs the homologous recombination function [3]. The BRCA1 p. L63* (NM_007294.4 (*BRCA1*): c.188 T > A(p.Leu63Ter)) variant is reported as pathogenic in the NIH ClinVar database [5]. A report suggested sensitivity to platinum agents regarding the treatment sensitivity of BRCA1 p.L63* variant [6]. Conversely, no reports have described the clinical sensitivity of PARP inhibitors to this variant, although the sensitivity of this variant to PARP inhibitors using in vitro patient-derived organoids is indicated [6].

An international phase III clinical trial (SOLO-2 trial) revealed that maintenance therapy with the PARP inhibitor Ola demonstrated a significant extension of progression-free survival (PFS) compared to placebo in patients with platinum-sensitive recurrent ovarian cancer with germline *BRCA1/2* variants [7]. Investigator-assessed median PFS was significantly longer with Ola (19.1 months, 95% confidence interval [CI]: 16.3–25.7) than with placebo (5.5 months, 95% CI: 5.2–5.8; hazard ratio [HR]: 0.30, 95% CI: 0.22–0.41, p < 0.0001).

Similarly, the ENGOT-OV16/NOVA trial revealed that the PARP inhibitor, niraparib, demonstrated a significant extension of PFS compared to placebo as maintenance therapy in patients with platinum-responsive recurrent ovarian cancer, regardless of BRCA variant status [8]. Patients in the niraparib group had a significantly longer median PFS than in the placebo group, including 21.0 vs. 5.5 months in the gBRCA cohort (HR: 0.27; 95% CI: 0.17–0.41). Our case, for the first time, represents a presumed OCC of CUP with the BRCA1 c.L63* germline variant, which was sensitive to both platinum-based chemotherapy and Ola in clinical practice.

Cases of HBOC syndrome reported as a CUP are extremely rare, and a literature search demonstrated only one reported case from China. A 63-year-old female patient presented with an egg-sized lump in her left iliac fossa with no primary sites. This case demonstrated an embryonic cell variant of BRCA1 (R71K), as well as sensitivity to PTX plus CBDCA, followed by Ola in maintenance therapy, after a tumor resection in the left iliac fossa [9]. This patient survived over 100 months from the onset, and the maintenance treatment with Ola was continued over 16 months in the literature. The patient in our case survives for > 30 months, and the maintenance treatment with Ola was continued for > 25 months.

HBOC syndrome is known to have low penetrance for ovarian cancer unlike familial adenomatous polyposis for colorectal carcinoma. Penetrance estimated by age of 70 years for ovarian cancer was 48.3% and 20.0% for *BRCA1* and *BRCA2*, respectively [10]. However, polyps develop in the early teenage years and result in a nearly 100% lifetime risk of colorectal cancer in cases of familial adenomatous polyposis [11]. Some ovarian cancers in HBOC syndrome may have occult cancer phenotypes. The prevalence of occult ovarian and fallopian tube cancers has been reported at the time of RRSO to be 2.3–23.5% [12]. Low penetrance of ovarian cancer may be responsible for CUP in some cases. Eight (7.8%) occult fallopian tube carcinomas (5 in tubal fimbriae only, 1 in tubal isthmus only, 2 in fimbriae and ovary) were detected in RRSO specimen from 102 women with BRCA genes variants [13]. As ovarian cancer only was 3 (2.9%), existence of the occult fallopian tube carcinomas should be taken care rather than ovarian cancer.

In the future, it is possible that the occult primary lesion would be identified more consistently if RRSO is actively carried out.
